# Supplementary material for: Are carbon emissions trading and green financial instruments synergistic? -Comprehensive quantitative research based on content analysis
Source: PLoS One. 2024 Mar 7;19(3):e0298601. doi: 10.1371/journal.pone.0298601 (PMC10919601; doi:10.1371/journal.pone.0298601)
Supplement: S1 Data — (ZIP) [file pone.0298601.s001.zip › Data/地级市Z_F工作报告.zip!/╣π╢1⁄2/╜¡├┼╩╨/└┤╫╘╣1⁄2╓┌║┼í╛┬φ┐╦╩2╛▌═°í┐_2005.docx]

各位代表：

　　我代表市人民政府向大会作政府工作报告，请予审议，并请市政协委员和各位列席人员提出意见。

２００４年工作回顾

　　２００４年是我市加快发展的一年。市政府在省委、省政府及市委的正确领导下，在市人民代表大会及其常委会的监督、支持下，全面完成了市委十届二次全会和市十二届人大三次会议确定的各项目标任务，经济建设和社会发展呈现新局面。

　　——经济发展跃上新台阶

　　全面实现各项工作目标。经济运行呈现出发展提速、结构优化、效益提高的良好态势。全市生产总值完成８３４．５６亿元，增长（与上年同比，下同）１２．２％，创８年来的新高，人均ＧＤＰ突破２５００美元；三大产业结构比例调整为８．９：４９．５：４１．６；地方财政一般预算收入完成２８．９４亿元，按可比口径计算，增长１８．４１％，增幅创近１０年的新高；全社会固定资产投资完成１９７．８８亿元，增长２２．８２％；实际吸收外商直接投资５．１２亿美元；外贸出口总额５０．７４亿美元，增长３８．９％；社会消费品零售总额３１１．５７亿元，增长１２．１％；城镇居民人均可支配收入１１９５８元，增长８．２６％；农村居民人均纯收入５１５９元，增长４％；城镇登记失业率２．９％；居民消费价格指数上升２．５％，保持基本稳定。各项主要指标的完成和超额完成，为今年全面实现″十五″计划奠定了坚实基础。

　　制造业竞争力进一步增强。全市规模以上工业增加值完成２９９．７６亿元，增长１８．３７％。新增固定资产投资备案工业项目１５８５个，投资总额１４１亿元，增长２８％。继制定鼓励发展信息产业的政策后，又出台了鼓励发展汽车零部件制造业和珠宝加工业的政策，我市成为全国最大的摩托车制造基地、全省重要的汽车零部件出口基地和珠宝加工基地。华尔润玻璃、大冶摩托车、益胜浮玻二期、潮连钻石岛恒和珠宝及中裕工业园等重点项目相继投产。新增麦克风、新材料、纺织化纤、电子信息等４个国家级产业基地，新会银洲湖区域被省命名为石化基地。中油碧辟石油有限公司、大长江集团、新会中集等３家企业跻身中国外资企业５００强。“莲花”牌白砂糖、“ＳＡＰ”牌铝型材新获中国名牌产品称号。技术改造投资完成２１．８２亿元，增长２１．９％。新增高新技术企业２４家，累计１２８家；高新技术产品产值３８０．５亿元，增长２９％；高新技术产品产值占工业总产值的比重为２２．５％，提高３．２个百分点。

　　招商引资取得重大突破。举办了机博会、牛仔服装节、水暖卫浴设备展销会和麦克风及电声器材国际展销会等活动，组织小分队赴澳洲、美加、欧洲、日韩等地招商。新批外商直接投资项目４４７个，合同外资金额１０．１２亿美元；新批和净增资１０００万美元以上的项目３７个，比上年增加８个，国内最大的利用外资零售项目中油碧辟石油有限公司正式落户我市。对欧盟、美国等市场出口强劲，分别增长３９％和５６％；出口商品结构进一步优化，机电产品出口占出口总额５６％。

　　民营经济快速发展。新增私营企业２６６１户，新增注册资金２９．３８亿元；民资投资额５７．１３亿元，增长６４％。规模以上民营工业总产值４６８．４４亿元，占全市规模以上工业总产值３５．４１％。个体和私营经济实现税收１６．０１亿元，增长２１．７５％。新增民营出口企业１９０家，民营企业出口额１２．４８亿美元，增长８７．３４％。

　　第三产业稳步发展。第三产业增加值完成３４７．４４亿元，增长１１．２％。旅游业持续增长，全年接待游客５４０万人次，其中国际游客２８万人次，增长２４％；旅游总收入４６亿元，其中国际旅游收入９２５４万美元，增长２１％。按五星级标准建设的逸豪酒店建成营业。现代物流业有新进展，辐射粤西地区的大中型货柜车进出境车检场建成启用，年通关处理能力８．５万辆次以上。

　　——“三化”建设取得新成效

　　各项扶持“三农”政策得到落实。我市各级财政一般预算投入农业农村２．７１亿元，增长１０．２％；免征农业税６３９８万元，农民人均减负２５元；安排种粮直接补贴资金９９１万元；发放农业机械购置补贴专项资金９８．８万元，粮食收购价增长两成以上，有效调动了农民生产积极性。解决农村富余劳动力转移就业新增１．２８万人。

　　农业产业化建设取得显著进展。农业龙头企业增至１５０家，获得国家级绿色食品认证产品１６个，省级无公害农产品生产基地发展到１０１个。制定并实施鼓励发展农民专业合作经济组织的政策措施，涌现一批体制灵活、服务能力强的新型农业合作经济组织。农产品出口创汇能力明显增强，水果出口值跃居全省第二位。

　　镇域经济发展步伐加快。制定并落实加快镇域经济发展的决定及相关政策措施，扩大了镇级经济管理权限，进一步完善了激励型财政体制，建立了市直有关部门调研联系镇（街）制度，推动了镇级经济加快发展。镇级财政收入１１．５亿元，增长２３．１％，占全市地方财政一般预算收入４０％。在全市８４个镇（街）中，７８个镇（街）财政收入实现增长，其中增幅超２０％的有５０个。专业镇和特色产业发展迅速，１１个省级专业镇实现工业产值６８３亿元，增长２３％。

　　——体制改革取得新进展

　　经济体制改革深入推进。成立市国有资产监督管理委员会，在深化改革中加强对国有资产的监管。市直国企完成改制累计１９９户，改制面为９３．９％。改善金融服务，成立市金融服务办公室，举办金融产品展销会和金融信息发布会，解决恩平市企业结算难问题。组建市政府投资工程建设管理中心并规范运作。进一步完善粮食流通体制改革。继续深化财政管理制度改革，市本级９０个部门、２０８个预算单位全部纳入了部门预算编制范围；强化财政国库集中支付；政府采购规模进一步扩大，平均节约率达１２．３％。

　　社会事业改革力度加大。出台了深化文化体制改革、建设文化名市的决定及配套政策。在市中心医院、五邑图书馆和市九中进行事业单位人事制度改革试点工作。完成市直财政拨款四所幼儿园的改革。继续推进医疗体制改革，民营医疗机构有序发展。

　　——城乡环境和建设上新水平

　　基础设施建设项目进展良好。台山发电厂１、２号机组和新会双水电厂两台机组投入营运。滨江大道、江珠高速、江中高速、江鹤高速二期、新会港二期等重点工程建设和广珠轻轨江门支线前期工作按计划推进。劳龙虎水道整治工程动工，崖门出海航道整治获省正式立项，并被列入省重点工程。

　　成功创建国家环保模范城。以创建国家环保模范城为抓手，实施“蓝天碧水”工程。水环境综合整治力度加大，江门河、天沙河、紫水河等河涌综合整治取得阶段性成果，建成丰乐污水处理厂和新会龙泉污水处理厂，西江江门段、潭江等主要河流水质保持较好水平。空气环境质量总体维持国家二级标准，市区全年空气质量日报优良率为９８．９％。以ＢＯＴ方式建成江门市医疗废物处理中心，实现全市医疗废物安全处理。完成市区生活垃圾处理场扩容，改造了新会西坑生活垃圾填埋场。全市２４３万亩生态公益林保护良好，开平市成为全省首个县级国家园林城市，鹤山市进入全省首批生态县之列，台山市北峰山、开平市潜龙湾分别获批为国家级和省级森林公园。

　　城乡建设协调推进。２６项市区城建重点项目进展顺利，完成投资额５．６亿元。院士路落成，会展中心建成使用，江礼大桥改造扩建即将竣工。滨江新区规划进行了国际招标。江门市区城市总体规划通过市人大审议。委托北京大学进行江门市可持续发展战略规划的研究取得初步成果。大力推进城镇化建设，新撤并３个镇，撤并率累计达２７％；调整撤并５５５个村委会，撤并率为２６．１％；５８条村完成了“村改居”工作；４个中心镇规划批准实施，另有４个中心镇规划修编通过初审。

　　——社会各项事业取得新进步

　　十项民心工程扎实推进。首次举办慈善公益“万人行”活动，募得捐款７８２５万元。把解决群众行路难、看病难、就学难、饮水难、住房难等问题列入市政府督办重点。镇通村公路硬底化建设完成１３５．４公里，受益群众２５万人。制定并实施《江门市区区域卫生规划》，公共卫生疾病预防控制体系、卫生执法监督体系及医疗救治体系建设不断加强。积极推进新型农村合作医疗，全市参加农村合作医疗人口１６９．９７万人，覆盖率为７１．４２％。落实教育扶贫工程，减免近２万名农村低收入家庭子女基础教育阶段书杂费，基本解决了农村困难家庭子女“读书难”问题；资助城镇困难中小学生２千多人次，对家庭经济困难高中学生实施资助政策。制定农村饮水安全建设规划，加快落实解决农村饮水难方案。继续推进安居工程，困难群众居住环境得到改善。大力清理解决建设领域拖欠工程款和农民工工资以及拖欠征地补偿款问题。抓好城乡水利防灾减灾工程，完成江新联围、天沙河江咀水闸和大隆洞水库等一批水利加固工程。成立食品安全委员会，加强食品卫生安全工作。强化安全生产监督管理，落实层级责任制，消除了一批安全事故隐患。

　　群众生活得到进一步保障。扶贫工作取得新成效，又有１１０条集体年纯收入３万元以下的村实现脱贫。社会保险覆盖面不断扩大，企业养老、失业保险缴费率分别达９６．９１％和９６．７７％。设立街道（镇）、社区劳动保障工作机构，新增２．６５万个就业岗位，１．０７万下岗失业人员实现再就业。残疾人康复就业工作成绩显著。对定恤定补重点优抚对象６７１６人发放资金１６４５万元，对城乡最低生活保障对象５．１万人发放低保救济金２７８４万元。

　　文化名市建设创品牌。深入开展“爱国、守法、诚信、知礼”现代公民教育活动。成功举办首届华人嘉年华活动，在海内外产生广泛影响。东湖广场被评为“全国特色文化广场”和广东省“十佳文化广场”。评选出全市哲学社会科学优秀成果。组建江门职业技术学院，扩大高等教育办学规模；完成中小学布局调整，改造１０８所老区、山区学校；江门一中新校区建设进展顺利；普及高中阶段教育步伐加快。新会区会城街道办事处成为省级教育强镇。大力推进全民健身活动，创建体育先进社区工作初见成效；举办亚洲高尔夫球精英赛、国际男子网球系列赛获好评。

　　其他各项事业取得新成绩。计划生育工作巩固提高，人口出生率下降０．０５个千分点，自然增长率降至３．０１‰，全面完成了省下达的人口计划任务。社区建设进一步加强，解决了一批社区居委会办公用房，建设和完善社区服务中心，依法开展社会救助管理工作，建成“星光老年人之家”７６个。人防１０１工程动工。外事、侨务、台湾事务工作有新进展。新闻、出版、档案、地志、宗教、老龄、气象等各项事业有新进步。

　　——民主法制建设和政府自身建设水平有新提高

　　自觉接受市人大及其常委会和市政协的监督。认真执行市人大及其常委会的决议。坚持重大问题听取市人大和市政协的意见。进一步完善办理人大建议、政协提案工作网络，全年办理人大代表建议１６１件，办理结果满意率为９５．６％；办理政协提案１５４件，办理结果满意率为９７．４％。对市长约见人大代表所了解的意见和市政协《社情民意》反映的问题，逐一督办并反馈。

　　依法行政工作全面推进。贯彻落实国务院《全面推进依法行政实施纲要》。组织实施《行政许可法》，全面清理审批事项，取消行政许可事项２３项，转按一般业务管理７８项。行政执法队伍建设和行政执法监督进一步加强。修订了《江门市人民政府工作规则》。加大法律援助力度。依法开展对政府采购、国库集中支付、部门综合预算、农业发展资金等重点事项的审计工作。

　　政府服务水平进一步提高。建立和拓宽政府与市民联系渠道，广泛听取各方面意见。建立全市经济形势分析联席会议和经济社会发展顾问咨询制度，广集良策，提高政府决策水平。从制度创新和加强管理上进一步改进机关作风，在市直窗口部门有序导入ＩＳＯ质量管理体系。加强政府目标管理考核督办工作，完成或基本完成１５３项年度考核目标任务。在政务公开工作中推广“群众点题”的做法，开设网上“侨乡论坛”。“１２３４５”政府服务热线扩至市直１９个部门和三区，共受理电话１０８１５次，广受市民好评。开展纠正损害群众利益不正之风的专项治理工作，查处了一批违纪违法案件，政府廉政建设和反腐败工作扎实推进。群众对市直部门的投诉较上年减少１５１１宗，下降５１．６％，其中下半年比上半年减少４０．７％。

　　社会大局保持稳定。深入开展严打整治斗争，重点打击“两抢两盗”、“六合彩”、非法传销等犯罪活动。坚决打击“法轮功”等邪教组织。进一步完善社会治安防控网络，创建平安社区１４０９个。深入开展缉毒专项行动，禁毒０３工程通过省的验收。监狱、劳教工作有新进步。健全和落实信访工作制度，妥善处理人民内部矛盾，维护了广大人民群众的根本利益。

　　各位代表，回顾过去的一年，我市经济和社会发展取得了可喜的成绩。这是贯彻落实党的十六大精神、“三个代表”重要思想和科学发展观的结果，是全市人民和中央、省驻我市单位及驻军部队团结奋斗的结果，是各民主党派、社会各界人士、外来投资者和建设者、港澳台同胞、海外侨胞关心支持的结果。在此，我代表市人民政府，向关心支持江门经济社会发展的同志们、朋友们、同胞们表示崇高的敬意和衷心的感谢！

　　与此同时，我们要清醒地看到我市经济社会发展中存在的问题，主要是：经济总量还不大，质量还不高，竞争力还不强，粗放发展模式还没有根本转变；影响社会稳定的因素增多，一些地方社会治安问题仍较为严峻；一些政府部门工作人员的工作作风、办事效率和服务质量需要改进；一些行业的收费成为市民投诉的热点等。对这些问题，市政府高度重视，正采取切实措施加以解决。

２００５年工作任务

　　２００５年是全面实现“十五”计划目标，衔接“十一五”规划的重要一年。我们面临的形势总体上是机遇大于挑战。我们必须增强发展的责任感和紧迫感，正视当前标兵越来越快、追兵越来越近的发展形势，充分发挥我市资源丰富的比较优势、基础设施不断完善的区位优势、华侨华人众多的侨乡优势，调动方方面面积极性，实现“科学发展，后发争先”的目标。

　　２００５年政府工作的指导思想是：以邓小平理论和“三个代表”重要思想为指导，以科学发展观统揽全局，认真贯彻落实党的十六届四中全会、中央经济工作会议、胡锦涛总书记视察广东重要讲话和省委九届六次全会、市委十届四次全会精神，继续实施工业强市、民资外资富市、科教兴市、环境优市战略，以调整优化产业结构，转变经济增长方式，提高产业竞争力为核心；以提高招商引资水平，推动内外源经济发展为动力；以区域互补，城乡协调，加强薄弱地区发展为重点；以加强城乡规划建设管理，营造优美生态环境为载体；以建设文化名市、文明城市，构建和谐社会为保障，推进经济社会全面协调可持续发展，全面实现“十五”计划目标，努力建设富强江门、和谐江门、绿色江门。

　　今年我市经济社会发展的主要预期目标是：生产总值增长１１．５％，地方财政一般预算收入增长１２％，固定资产投资增长１３％，外贸出口总额增长８％，社会消费品零售总额增长１２％，居民消费价格涨幅控制在３．５％左右，城镇登记失业率控制在３．５％以内，人口自然增长率控制在５．６‰以下，城镇居民人均可支配收入实际增长８％，农村居民人均纯收入实际增长４％左右。

　　今年政府工作要着力抓好以下个方面：

　　一、调整优化产业结构，加快经济增长方式的转变

　　以港口经济和大项目带动为重点，推动工业结构向适度重型化转变。以银洲湖和广海湾为重点区域，发挥新会港、台山广海港的港口优势，加强基础设施和配套设施建设，大力发展港口经济。充分利用银洲湖列入省石化基地的有利条件，加快发展石化中下游产业；积极发展高增长性、产业链条长的汽车零部件产业和装备制造业；抓好亚太纸业、名冠、摩迪等项目建设以及大长江、维达、华泰、华尔润等项目的扩产扩建，提高重化工业占工业总产值的比重。大力实施名牌带动战略，力争再创２个中国名牌产品和驰名商标，新增５个省名牌产品。

　　以加快新兴产业发展为重点，推动工业向高新技术主导的方向转变。大力发展以高新技术为龙头的新兴产业，大力推动以高新技术改造传统产业，大力扶持发展科技含量高的潜力产业。以省的产业规划为指导，立足于发挥比较优势，延伸产业链和占领行业制高点，制定加快电子信息、摩托车和汽车零部件、石化加工、精密机械、纸业、纺织服装、建材加工、钻石珠宝加工等产业发展的规划，形成特色鲜明、布局合理、结构优化、层次提升的发展格局。

　　以完善技术创新服务体系为重点，推动传统产业和中小企业向技术密集、集群发展转变。鼓励传统产业依靠技术进步提高核心竞争力。集中工业发展资金重点抓好技术创新服务平台、质量技术标准和检测检验服务平台、信息服务平台建设，为中小企业加快发展创造更好的环境，增强产业集聚吸引力，提高产业集群发展水平，做大做强９个国家级制造业基地和其他特色产业基地。

　　以节约用地、节约能源、节约用水和保护环境为重点，推动经济增长方式向节约型经济和绿色经济转变。按照减量化、再利用、资源化的原则，坚持开发与节约并举，推动企业实施节能、节水和新型环保技术，推行清洁生产；构建节约型产业结构和消费结构，建设节约型社会。严格按照用地投资控制标准，提高工业用地使用效益。积极做好把新会双水拆船钢铁有限公司、银洲湖纸业基地、甘化股份有限公司纳入国家循环经济示范点的工作。

　　以大力发展现代服务业为重点，推动产业结构向一、二、三产业协调发展转变。抓住泛珠三角战略、ＣＥＰＡ实施的历史性机遇，充分利用粤港物流合作平台，加快我市制造业、商业与香港物流业的对接，鼓励在我市设立拓展第三方物流的节点与基地。选择开阳、江中、江珠等高速公路沿线和新会港、台山广海港等，规划建设若干个现代物流基地。采用现代物流管理技术和装备，建立物流信息平台，为企业提供采购、加工、包装、储运、配送和进出口代理等服务。发展会展经济，打造会展品牌。结合举办首届广东国际旅游文化节分会场活动，继续办好侨乡旅游节，完善旅游配套设施，整合旅游资源，提高旅游业发展水平，使江门五邑成为省内主要旅游胜地之一。培育消费市场，拓展汽车、住房、教育、娱乐、休闲、体育、健身等消费领域，发挥现代服务业对经济增长的拉动作用。

　　二、加大招商引资力度，增创改革开放新优势

　　抓住契机，提高招商引资水平。充分利用中国投资环境论坛和粤台经济技术贸易交流会在我市举办的机遇，结合办好机电博览会，在扩大引资规模的同时，更加注重提高引资质量。发展与香港和澳门的长期经贸战略伙伴关系，加强与世界银行、香港工贸机构的紧密合作，增进与外国驻华机构、华侨社团、国内外商会和行业协会、国家及省有关部门的沟通联系，扩大招商网络。加大以侨引商、以商引商的力度，发挥中介招商、代理招商的作用，建设一支业务熟、外语精、作风好、干劲足的高素质专业招商队伍，努力实现招商引资新突破。

　　落实招商引资责任制，突出抓好产业招商、异地招商和增资扩产。坚持主要领导亲自抓招商、亲自联系重大招商项目、亲自协调解决招商中的难题，开创招商工作新局面。抓好全程跟踪服务特别是引进后服务，切实为投资者排忧解难，为企业增资扩产创造条件。加强招商引资的针对性，以美加、欧洲、日韩等地区为重点，开展以小分队为主的对口招商、产业招商和择优招商。建立和完善“本地招商、异地办厂”的利益共享机制，促进热点地区与偏远地区招商互补，实现山区镇和偏远镇招商引资的重大跨越。

　　进一步优化投资和营商环境。全力争取台山直供电试点，营造招商引资新“洼地”。深入开展“人人关系投资环境，事事影响江门发展”的责任意识教育。突出抓好知识产权保护，切实保障投资者权益。推行项目手续全程代办制度，提高对投资者的服务水平。完善企业信用信息网建设，维护重诺守信的营商环境。坚持效益、特色、集约的原则，加大对重点园区的投入力度，完善基础设施配套，提高产业园区的承载力和吸引力。

　　优化外贸结构，提高应对国际贸易摩擦能力。采取积极措施努力减轻现行出口退税机制对财政产生的沉重压力。优化出口商品结构，推动加工贸易升级。实施电子报关和企业联网监管，提高通关效率。抓紧落实入世后过渡期的应对措施，开展防范技术性贸易壁垒及反倾销的培训，加强联合行动。鼓励有条件的企业“走出去”，开拓更广阔的国际市场。坚决打击出口骗税行为。

　　深化各项体制改革，进一步改善金融服务。既要坚定不移地深化国有企业改革，又要妥善处理关系职工切身利益的问题。健全公司法人治理结构，落实国有资产出资人职责。规范国有企业改制重组、产权交易和重大投资行为，建立国有资本经营预算制度。深入推进行政审批制度改革，加快政府职能转变。把握农村信用社改革机遇，建立金融服务平台，健全信息发布会制度，加强银企、银地沟通，完善贷款担保机制和企业信用保障机制，积极发展民间贷款信用担保机构。

　　三、坚持把“三化”解决“三农”摆在经济工作的重要位置，增强镇域经济实力

　　多管齐下，加大支农力度。今年我市各级财政一般预算安排农业方面的支出３．３１亿元，增长２１．９８％。全面落实种粮补贴、农机补贴、粮食最低收购价、农资价格监管、应急种子储备、地力培育等政策措施。提高农业综合生产能力，以加强农田水利建设为重点，加大农业基础设施投入力度，高质量建设好农业标准化示范区，实现全市良种覆盖率９５％以上，整治改造４万亩低产田。鼓励创农业品牌。加强对农村劳动力转移就业培训，推行免费“定单培训”。建立农村社会保障制度和农村社会救助体系。继续实施扶贫工程，改善贫困农户的基本生产生活条件。今年集体纯收入３万元以下的村再脱贫５０个以上。

　　大力发展特色产业，提高工业化水平。完善镇级工业发展规划，培育、发展特色产业，建立企业、行业和专业镇品牌体系，做强专业镇，提高农村工业化水平，增强工业反哺农业能力，加快镇域经济发展。今年财政收入超千万元的镇（街）增加５个。

　　以加强中心镇建设为重点，加快城镇化进程。坚持以城带乡，提高城镇规划建设管理水平。年内要完成中心镇规划修编；全面落实省委、省政府赋予中心镇的十项政策；加强中心镇工业载体和基础设施、生活配套设施的建设，吸引生产要素集聚，发展第三产业，增强吸纳农村劳动力就业能力。取消对农民工进城的限制和不合理收费，优化农民工就业环境。

　　加强对农业产业化的服务。推广台山供销社的经验，吸收农民参股入社，充分发挥供销社为农服务的功能。积极发展农民专业合作经济组织，建立农村专业协会，提高农民的组织程度，为农业产业化奠定扎实基础。发展高效益和外向型农业。增强３０家农业产业化龙头企业的辐射带动能力。完善农业科技推广服务体系、农产品质量安全检测网络及动植物防疫检疫体系，推进农业科技进步。搞好中心市场、专业批发市场和主产区产地市场建设，确保农产品销售渠道通畅。

　　四、继续狠抓基础设施建设，提高城乡建设水平

　　突出抓好交通能源重点项目的规划建设。积极做好我市交通路网与港珠澳大桥路网、广珠铁路、珠三角城际轻轨的衔接工作。争取“十一五”规划属国家和省审批的交通能源重大项目尽早立项。全力配合江中和江鹤二期高速公路今年建成通车，江珠高速公路明年通车，江肇高速公路今年动工。高标准规划滨江新区，确保滨江大道完成年度建设计划。抓紧开展崖门电厂前期准备工作；大力支持台山电厂３、４、５号机组建设；加快推进台山风力发电项目和西江电厂技改项目；千方百计做好电力供应保障工作。落实崖门出海航道和劳龙虎航道整治疏浚的保障措施。

　　提高城市建设和管理水平。抓好市区２７项城建重点项目建设。有计划、分步骤完善市区道路、排水、绿化、环保等基础设施功能，提高人居环境水平。高质量完成市区河南片排水管网、文昌沙水质净化厂二期和天沙河治理工程等项目的世界银行贷款前期工作，确保通过初评。改革市政管理方式，实行市政设施维护运行市场化，提高政府资金使用效益。

　　继续加大环保工作力度。以建设国家生态市为目标，编制江门市环境保护规划及专项规划。全面实施“碧水、蓝天、洁净、宁静”工程，市区突出抓好天沙河江门段生活污水截污管网建设；各市、区要确保完成重点河段、河涌的整治任务；要按期建成台山、开平、鹤山、恩平的城市污水处理厂和１２个中心镇的生活污水处理厂；巩固、提高工业污染源治理达标成果，坚决查处偷排行为；加强对机动车尾气排放和饮食店油烟污染的监督管理。保护饮用水源，保障饮用水安全。筹建市区新的生活垃圾处理场。

　　五、切实解决人民群众关心的热点难点问题，大力构建和谐江门

　　构建覆盖面广、惠及全民的城乡公共卫生网络。进一步加强疾病预防控制体系、卫生监督体系、突发公共卫生事件应急救治体系建设；加快各市、区疾控项目建设进度；按期完成市突发公共卫生事件应急中心一期建设。加强重大传染病的防治，落实全国艾滋病综合防治台山示范区各项工作措施。健全新型农村合作医疗制度，巩固和提高农村合作医疗人口覆盖率。鼓励外资民资兴办综合医院或专科医院。加强医疗机构管理，严格规范医疗收费，完善药品采购集中招投标机制，切实降低医药收费。深入持久开展爱国卫生运动，继续推进农村改水改厕工作，进一步改善农村卫生环境。

　　推进人才战略，发展多层次、多元化的教育体系。完善“以县为主”的农村义务教育管理体制，完成５９所中小学布局调整工作，推进教育信息化工程，巩固提高义务教育质量。大力发展高中阶段教育，创建国家级示范性普通高中，加快教育强镇、强市（区）步伐。确保江门一中新校区今年新学年投入使用，江门职业技术学院新校区明年上半年落成。进一步办好五邑大学和各大中专学校。鼓励发展民办教育。规范“一费制”收费管理。加强人才工作，深化事业单位人事制度改革；大力培养、引进并用好各类管理人才、科技人才、经贸人才和高技能人才；健全各类人才选拔任用、考核评价和激励机制，营造有利于人才干事创业的环境。

　　加强就业工作和社会保障体系建设。努力扩大社会保险覆盖面，实施社会保险市级统筹，推行地税全责征收，实现参保人数和收缴率增长达标。加大就业岗位开发力度，全年新增２万个就业岗位；完善就业服务体系，加强职业技能培训和创业培训，鼓励运用小额担保财政贴息贷款自主创业。确保改制国企职工妥善安置。加强劳动保障监察和劳动关系协调工作，维护劳动者合法权益。建立机关事业单位工作人员个人养老保险账户制度、公务员医疗保险制度和事业单位失业保险制度。做好企业退休人员社区管理工作。抓好市区新劳动力市场、社会保险服务大厅和高级技工学校扩建等项目建设。

　　以创建省文明城市为契机，推进文化名市建设。广泛开展“爱国、守法、诚信、知礼”现代公民教育活动，加强大学生思想政治教育和未成年人思想道德建设，扎实、深入开展群众性精神文明创建活动。深化文化体制改革，发展民营文化企业，培育多元化的文化产业体系。发挥会展中心及其附属设施作用，力争年内完成华博馆、美术馆筹建工作。加强宣传侨乡文化，大力打造碉楼、人文、旅游、社区和广场文化品牌；发扬民间艺术文化；保护开发崖门古战场。

　　体察民情，紧贴民心，多办实事。继续实施“十项民心工程”，帮助困难群众解决就业、就医、子女上学、住房、打官司等方面遇到的问题。抓好城乡水利防灾减灾工程、农村饮水工程和以解决水库移民住房为重点的农村安居工程建设。着力整治教育、医疗、药品等热点价格问题和侵犯消费者权益的问题，开展食品药品安全专项整治行动。适当提高公务员和教师的收入水平。建设市区体育公园，推动全民健身活动，增强市民体质。

　　加强安全生产。落实安全生产责任制，严格监督和管理安全生产，特别是对重点行业、重点企业的安全生产监管，坚决整改安全隐患，严防重特大安全事故发生。落实消防安全和道路交通安全措施，筹建市区消防特勤大队。

　　切实维护社会稳定。充分发挥人民调解、行政调解和司法调解的作用，建立和完善社会矛盾化解机制。认真执行《信访条例》，加强和改进信访工作，健全信访工作联席会议制度；市直涉民部门及各市、区、镇（街）要建立领导定期接访制度；对信访突出问题及两次以上的信访问题，要作重点检查和督办。切实解决拖欠农民征地款和农民工工资问题。严密防范和严厉打击境内外敌对势力、暴力恐怖势力和“法轮功”等邪教组织的破坏活动。加强社会治安综合治理，完善城乡社会治安防控体系，坚决打击以“两抢两盗”、赌博、制毒贩毒为重点的违法犯罪活动，加快推进创建无毒社区和平安社区活动，建设平安、和谐的江门。

　　促进社会各项事业协调发展。做好双拥共建和优抚安置工作。开展全民国防教育，提高国防动员建设和民兵预备役工作水平。落实人口与计划生育法，稳定低生育水平，提高出生人口素质。发展妇女儿童事业。做好新形势下侨务、外事、台湾事务工作，推进新闻、出版、人防、气象、残联、宗教、老龄、档案、地志等工作。

　　六、提高执政能力，建设以民为本的服务型政府

　　提高依法行政水平。认真贯彻《全面推进依法行政实施纲要》，严格依法行使权力、履行职责，规范政府行政行为；加强法律学习，增强政府工作人员的依法行政观念，严格依法办事，建设法治政府。自觉接受人大及其常委会的监督，切实办好人大代表议案、建议和政协委员提案。提高政府的社会管理能力，建立各种突发事件应急机制，保障社会公共安全。严格执行政府工作规则，进一步精简会议和文件，改进机关作风，提高行政效率。

　　提高民主科学决策水平。坚持经济形势分析联席会议和经济社会发展顾问咨询制度。完成江门市可持续发展战略规划和“十一五”规划的编制工作。搞好经济普查。健全重大决策和重要规划的论证和听证程序，定期听取人大代表和政协委员的意见建议，广泛了解社情民意，加强决策的民主性和科学性。提高生财理财水平。加强对财政工作的领导，确保财政收入稳定增长，提高地方财政一般预算收入占ＧＤＰ的比重。进一步完善激励型财政管理体制，理顺各级财政关系，调动各方面发展经济的积极性，增强各级财政实力。调整优化支出结构，优先保证公共财政支出，提高资金使用效益。增强财政收支透明度，建立有效的监控机制。举办镇长理财学习班，加强基层财政管理。

　　提高服务群众、服务企业、服务基层水平。坚持以人为本，执政为民，以开展保持共产党员先进性教育活动为动力，加强政府自身建设。以制度建设为重点，在市直机关全面导入ＩＳＯ质量管理体系，使每一个工作环节、每一个工作人员的服务效率和质量都有管理措施作保证。抓好以“群众点题”为重点的政务公开工作，加强电子政务建设，健全社会公示、社会听证制度，让市民更广泛地参与公共事务管理。进一步提高“１２３４５”政府热线服务水平，及时解决市民反映的热点难点问题，为市民提供“零距离”服务。深入基层、深入一线，坚持市领导挂钩扶持贫困村和职能部门挂钩联系镇（街）制度，务求“十百千万”干部下基层帮扶工作取得实效。发挥政府职能部门为企业服务平台的作用，主动、及时为企业排忧解难。加强机关作风建设考评工作，表彰先进、鞭策后进。认真核查对政府工作人员的投诉，坚决查处违纪违法人员。

　　提高廉政建设水平。坚持不懈地开展反腐败斗争，落实廉政责任制，规范各级领导干部从政行为，坚决整治损害群众利益的行业不正之风。严格执行建设工程项目招投标、经营性土地使用权出让、产权交易和政府采购等制度，从源头上防止腐败。

　　各位代表，江门正处于新的历史发展机遇期。让我们高举邓小平理论和“三个代表”重要思想伟大旗帜，全面贯彻落实科学发展观，在中共江门市委的正确领导下，以饱满的精神、扎实的作风，上下一心、团结一致，抢抓机遇、开拓进取，在统筹发展、协调发展中加快发展，为构建富强江门、和谐江门、绿色江门，把我市建设成为全面、协调、可持续发展的现代化大城市而努力奋斗！
